# Volatile Chemistry and Aroma Differences in Commercial Soy Sauces: Sensory Links and Odorant–Receptor Interactions

**DOI:** 10.3390/foods15193545

**Published:** 2026-10-04

**Authors:** Kun Zhao, Qinguo Quan, Ping Wang, Siqi Hu, Baoguo Sun, Dandan Pu

**Affiliations:** 1 China Key Laboratory of Geriatric Nutrition and Health, Ministry of Education, Beijing Technology and Business University, Beijing 100048, China; 15670576215@163.com (K.Z.); qinguo_quan@163.com (Q.Q.); wp1391267159@163.com (P.W.); sunbg@btbu.edu.cn (B.S.); 2 Jiangxi Province Key Laboratory of Sustainable Utilization of Traditional Chinese Medicine Resources, Institute of Traditional Chinese Medicine Health Industry, China Academy of Chinese Medical Sciences, Nanchang 330115, China

**Keywords:** soy sauce, headspace gas chromatography–ion mobility spectrometry, aroma compounds, sensory evaluation, molecular docking

## Abstract

Volatile and sensory differences among 28 commercial soy sauces were investigated using HS–GC–IMS, check-all-that-apply (CATA) evaluation, aroma addition experiments, and molecular docking. Ninety-nine volatile compounds from 14 chemical classes were detected. Total semiquantitative volatile concentrations ranged from 2024.56 to 6279.59 μg/mL, with esters, alcohols, and aldehydes accounting for 70.68% of the total. Of the 24 CATA descriptors, 13 were retained for product-level correlation analysis. Exploratory Pearson analysis indicated that alcoholic, savory, and malty attributes were associated with a group dominated by fatty acids, branched-chain alcohols, and esters, whereas woody and chocolate-like attributes showed an opposing pattern involving several aldehydes, ketones, and alkylpyrazines. Fifteen candidate compounds were evaluated by single-compound addition. Benzaldehyde and ethyl propanoate enhanced savory perception; 2-ethyl-3,5-dimethylpyrazine enhanced the alcoholic attribute; methyl 2-methylbutyrate enhanced smoky, meaty, and alcoholic attributes; and ethyl acetate enhanced malty, meaty, and alcoholic attributes. Several other additions produced opposing or nonsignificant effects, indicating matrix- and concentration-dependent responses. Among the binary docking models, guaiacol–2-ethyl-3,5-dimethylpyrazine and homofuraneol–benzaldehyde produced the lowest scores with OR10G4 (−10.10 kcal/mol) and OR5M3 (−13.32 kcal/mol), respectively. In combination, these results show that product-level correlations can guide candidate selection but require matrix-based sensory verification, while docking provides structural hypotheses for odorant co-binding.

## 1. Introduction

Soy sauce is a representative fermented condiment in East Asian cuisine and is mainly produced from raw materials rich in protein and starch through microbial fermentation [1]. During fermentation and subsequent processing, enzymatic hydrolysis, microbial metabolism, Maillard reactions, and thermal reactions together generate a complex aroma system composed of numerous volatile compounds [2]. Aroma is one of the most important factors that determine soy sauce quality, consumer preference, and market acceptance. However, commercial soy sauces differ substantially in terms of raw materials, fermentation processes, processing conditions, and product positioning, resulting in marked variations in their aroma profiles and sensory characteristics.

In recent years, techniques such as gas chromatography–mass spectrometry (GC-MS), two-dimensional gas chromatography–olfaction–mass spectrometry (GC × GC-O-MS), gas chromatography–olfaction–mass spectrometry (GC-O-MS), and headspace gas chromatography–ion mobility spectrometry (HS-GC-IMS) have been widely used to characterize aroma-active compounds in soy sauce. These techniques are often combined with pretreatment methods such as solvent-assisted flavor evaporation (SAFE), solid-phase microextraction (SPME), dynamic headspace sampling (DHS), and solid-phase extraction (SPE), aroma extract dilution analysis (AEDA), odor activity value (OAV), relative odor activity value (ROAV), aroma recombination, and omission experiments to identify key aroma contributors [3,4,5]. Previous studies have shown that sulfur-containing compounds, phenolic compounds, furanones, pyrazines, aldehydes, alcohols, and esters are important contributors to soy sauce aroma [6,7,8]. Methylthioacetaldehyde, HEMF, and 4-ethylguaiacol have been reported as representative compounds associated with strong aroma activity, caramel-like notes, and smoky notes [6,9]. Nevertheless, most existing studies focus on the identification of individual key aroma compounds, whereas the overall aroma perception of soy sauce is determined by the combined effects of multiple compounds rather than by a single compound.

Due to the complexity of the soy sauce matrix, analysis based solely on compound concentrations cannot fully explain the sensory differences among commercial soy sauces. Aroma compounds may exert additive, synergistic, or masking effects during perception, and these interactions can strongly influence the final aroma profile [2]. Therefore, it is necessary to establish a systematic relationship among volatile aroma composition, sensory perception, and aroma interactions. HS-GC-IMS provides a rapid and sensitive approach to generating volatile fingerprints of complex food matrices with minimal pretreatment, making it suitable for comparing aroma differences among multiple soy sauce samples [10,11]. Meanwhile, the CATA method offers a simple and reliable way to collect sensory data from trained panels. Mahieu et al. [12] and Jaeger et al. [13] demonstrated that CATA can effectively describe consumer-perceived sensory attributes provided that appropriate descriptive terms are used.

To further link instrumental data with sensory perception, statistical methods like Pearson’s correlation coefficient can be used to identify aroma compounds associated with specific sensory attributes. However, statistical correlations alone cannot confirm whether these compounds truly contribute to aroma enhancement, synergy, or inhibition. Therefore, addition experiments are needed to validate the sensory contribution and interaction patterns of selected aroma compounds. Moreover, molecular-level interpretation based on olfactory receptor (OR)–odorant interactions can provide further mechanistic insights into the molecular basis of aroma perception. Molecular docking has been increasingly applied to predict the binding modes, intermolecular forces, and binding affinities between aroma compounds and ORs [14,15,16]. For example, Wang et al. [17] reported that guaiacol can interact with OR1G1, OR2T1, and OR10G4 through hydrogen bonding and π–π interactions. In addition, high-throughput functional assays have shown that OR5M3 can be selectively activated by furanone and homofuraneol [18], suggesting that receptor-level analysis may help explain the perceptual contributions of characteristic aroma compounds in soy sauce.

The integration of instrumental and sensory data requires statistical procedures that account for data dimensionality, measurement scale, and multiple testing. Product-level correlation analysis can identify covariation between volatile compounds and sensory attributes but cannot establish that an individual compound directly produces or enhances a particular perception. In contrast, addition experiments assess sensory responses to defined compound doses within specific soy sauce matrices, and their outcomes may be influenced by background volatile composition, masking effects, and mixture interactions. Molecular docking represents a further computational level of analysis that can identify plausible receptor–ligand binding conformations but cannot demonstrate receptor activation or sensory causality. Accordingly, these approaches were interpreted as distinct but complementary levels of evidence rather than as a sequential validation framework. The major volatile classes and characteristic sensory descriptors of soy sauce have been extensively documented. Diez-Simon et al. summarized the contributions of acids, alcohols, aldehydes, esters, furanones, pyrazines, phenols, and sulfur-containing compounds to established sensory dimensions, including malty, caramel-like, alcoholic, smoky, and roasted notes [19]. More recently, Ji et al. integrated HS-SPME-GC-MS, chemometric analysis, relative odor activity value screening, and molecular docking to identify characteristic volatile constituents of Chinese geographical-indication soy sauces and predict their interactions with olfactory receptors [20]. Therefore, the novelty of the present study does not arise from the identification of these established chemical classes or sensory descriptors, nor from the general combination of volatile profiling and molecular docking.

Therefore, this study investigated differences in aroma characteristics and their potential underlying mechanisms among 28 commercial soy sauce samples. Volatile profiles were characterized using HS–GC–IMS, while sensory attributes were evaluated using the CATA method. Pearson correlation analysis was performed to examine the associations between volatile compounds and sensory attributes and to screen for candidate compounds potentially contributing to sensory differentiation. Subsequently, aroma addition experiments were conducted to evaluate the effects of selected compounds on sensory perception in representative soy sauce matrices. Finally, molecular docking of the selected compounds with their corresponding olfactory receptors was performed to explore the potential molecular basis of aroma perception. This study provides an integrated framework for understanding aroma differentiation and potential aroma perception mechanisms in soy sauce, thereby offering a scientific basis for flavor quality evaluation, product differentiation, and aroma modulation.

## 2. Materials and Methods

### 2.1. Materials and Reagents

A total of 28 commercial soy sauce samples were purchased from supermarkets in Beijing, China (Table 1). The samples represented different brands and product types, including light, dark, low-salt, seafood-flavored, organic, and Japanese-style soy sauces. Each sample was assigned a code for subsequent analysis. Based on the grades declared on the product labels, 23 samples were special grade, three were grade I, and two were grade II.

Organic compounds 2,4,6-Trimethylpyridine (99%), ethyl propanoate (≥99.5%), 3-methyl-3-buten-1-ol (98%), 2-methylpropyl acetate (≥99.5%), 2-ethyl-3,5-dimethylpyrazine (99%), butanal (reagent grade, ≥98.0%), isohexanol (98%), heptanal (≥98.0%), methyl 2-methylbutyrate (≥99.0%), ethyl acetate (≥99.5%), 1-pentanol (≥99.0%), 2-methyl-1-butanol (≥99.0%), (E)-2-hexen-1-ol (97%), benzaldehyde (≥99.5%), and (Z)-3-hexenol (≥98%, GC) were all purchased from Beijing Mairuida Technology Co., Ltd., Beijing, China.

### 2.2. The Check-All-That-Apply (CATA) Method Sensory Evaluation

The check-all-that-apply (CATA) method was used for sensory evaluation in this study. The sensory panel was established and trained in accordance with GB/T 16291.1-2012, Sensory analysis—General guidance for the selection, training and monitoring of assessors—Part 1: Selected assessors, which is equivalent to ISO 8586-1:1993 [21]. Thirty healthy, non-smoking volunteers with no history of rhinitis were recruited from the laboratory. After a series of training and screening procedures, the candidate assessors were evaluated in terms of descriptive vocabulary, sample discrimination ability, and evaluation stability. Finally, 14 assessors, including eight females and six males aged 23–27 years, were selected to participate in the subsequent sensory evaluation and complete the CATA questionnaire. All participants were fully informed of the experimental content and procedures and provided written informed consent. The CATA questionnaire included the following aroma descriptors: sweet, sour, savory, chocolate-like, sauce-like aroma, caramel, milk-like, meaty, fatty, roasty, smoky, spice, green, fruity, malty, mushroom, soybean-like, floral, woody, earthy, metallic, alcoholic, cabbage, pungent. All sensory evaluation procedures and related information were explained to the sensory panelists, and informed consent was obtained from all participants before the evaluation. The sensory evaluation experiment was approved by the ethics committee of Beijing Technology and Business University (Approval Code: BTBU202521; Approval Date: 12 March 2025).

Each sample (10 mL) was placed in a 50 mL colorless, odorless plastic bottle. The 28 samples were evaluated in four sessions of seven samples each. Samples were identified by randomized four-digit codes and presented in randomized order. Assessors completed the CATA task for every sample. Roasted and ground coffee beans were available between samples for olfactory reset, and all evaluations were conducted under the same laboratory conditions. The resulting data were retained only as product-level citation frequencies because the assessor-level binary records were no longer available.

### 2.3. Headspace Gas Chromatography–Ion Mobility Spectrometry (HS-GC-IMS)

Volatile compounds in the 28 soy sauces were analyzed by HS-GC-IMS. The internal standard, 2-octanol (1.744 mg/mL; 50 µL), was added to 0.5 mL of sample, and the mixture was incubated at 40 °C for 15 min. A 500 µL headspace aliquot was injected with a heated syringe into the HS-GC-IMS instrument (G.A.S., Dortmund, Germany). Separation used an MT200 column (30 m × 0.53 mm × 1 µm; Restek Corporation, Bellefonte, PA, USA) isothermally at 70 °C with nitrogen (99.9%) as carrier gas. The carrier flow was held at 2 mL/min for 2 min, increased to 5 mL/min over 8 min, to 100 mL/min over 5 min, and to 140 mL/min over 15 min, followed by a 1 min hold. The IMS drift tube was operated at 45 °C with a drift-gas flow of 150 mL/min, a 3H ion source (300 MBq), and a drift voltage of 6.5 kV.

### 2.4. Qualitative and Quantitative Analysis

GC-IMS data were processed using Laboratory Analysis View (LAV, Version 0.4.0) software. Compound identification was based on retention indices and relative drift times by comparison with the instrument’s GC-IMS database and, where available, authentic reference standards. Retention indices were calculated using 2-alkanone standards. Relative concentration equivalents were calculated from analyte-to-internal-standard peak-area ratios using 2-octanol (1.744 mg/mL; 50 µL) and were treated as semiquantitative. Each sample was analyzed in triplicate.

### 2.5. Addition Experiments

#### 2.5.1. Screening of Experimental Soy Sauce Samples

Based on the sensory results of CATA analysis among 28 soy sauce samples, one soy sauce with a relatively low selection frequency for each of the five target attributes (savory, smoky, malty, meaty, and alcoholic) was selected as the corresponding matrix. Each candidate compound was added to the selected matrix at a concentration equal to the difference in its measured concentration between a high-concentration reference sample and the selected low-concentration sample. Stock solutions of each target aroma compound were prepared by accurately weighing each compound and dissolving it in 10 mL of 10% aqueous ethanol. All stock solutions were freshly prepared before each test and stored refrigerated in sealed brown vials. For each addition assay, an appropriate volume of the corresponding stock solution was spiked into 10 mL of soy-sauce matrix. After gentle mixing, samples were equilibrated at room temperature prior to sensory evaluation, with detailed spiking amounts listed in Table 2. Five sets of sensory-addition tests were performed in total. Each test sample (10 mL) was transferred into a 15 mL tasting cup, coded with four-digit random blind labels and presented to panelists in a fully randomized order. The sensory panel consisted of 14 trained evaluators (6 males and 8 females), and each panelist assessed all test samples. Roasted and ground coffee beans were provided for olfactory reset between samples, and a 5-min mandatory rest interval was arranged between test groups to mitigate sensory fatigue and aroma carry-over effects. Panelists judged the changes in key aroma attributes to verify the sensory contribution of the supplemented volatile compounds.

#### 2.5.2. Screening for Target Volatile Aromatic Compounds

Candidate aroma compounds exhibiting strong correlations (|r| ≥ 0.7, *p* < 0.05) with at least one of these sensory attributes were initially identified. Subsequently, compounds with OAV > 10 in at least one product were retained where applicable, and the original exploratory, unadjusted correlation map was used only to assign OAV-screened candidates to target-attribute assays (Table S1). Because multiplicity was not controlled at that screening stage, the map was not treated as confirmatory evidence. OAV was calculated as the ratio of the semiquantitative concentration determined using the internal-standard method (Ci) to the odor threshold in water (OTi), as shown in Equation (1). The OAV > 10 cutoff was a pragmatic screening criterion intended to reduce uncertainty arising from aqueous thresholds and GC-IMS semiquantification, rather than a universal definition of sensory importance [22].

(1) OAV=Ci/OTi,

where Ci is the concentration of the aroma compound in the sample (mg/kg), and OTi is its odor threshold in water (mg/kg).

On this basis, OAV-screened compounds were assigned to the applicable target-attribute and soy-sauce-matrix combinations. The addition experiments were analyzed as exploratory, matrix-specific sensory interventions and were interpreted independently of the FDR-controlled product-level correlation analysis.

#### 2.5.3. Addition Experiments Sensory Evaluation

Following the sensory evaluation procedure described in Section 2.2, comparative assessments were conducted using a predefined evaluation form. The evaluation consisted of five independent attributes: savory, smoky, malty, meaty, and alcoholic. For each soy sauce sample spiked with a single aroma compound, a blind comparison was performed against the corresponding control sample. Evaluators recorded two judgments sequentially: first, whether a perceptible aroma difference existed between the test and control samples; and second, the intensity of change in the target aroma attribute (increase, ↑ or decrease, ↓).

### 2.6. Molecular Docking

Guaiacol and homofuraneol were selected as reference ligands for OR10G4 and OR5M3, respectively, based on previously reported receptor–odorant associations [17,18]. The accompanying ligands were selected from the compounds included in the addition experiments to construct binary systems containing different classes of aroma compounds. The three-dimensional receptor models were obtained from the AlphaFold Protein Structure Database: AF-Q8NGN3-F1 for OR10G4 and AF-Q8NGP4-F1 for OR5M3. Both were predicted full-length models without missing residues. Receptors were prepared using AutoDock Tools 1.5.7 by removing waters, adding polar hydrogens according to standard protonation states at pH 7.0, merging nonpolar hydrogens, assigning Gasteiger charges, and exporting the structures in PDBQT format. Three-dimensional ligand structures were retrieved from PubChem, converted to PDB format using Open Babel 3.1.1, protonated at pH 7.0, and energy-minimized. Rotatable bonds were defined, Gasteiger charges were assigned in AutoDock Tools 1.5.7, and the ligands were exported in PDBQT format.

Docking was performed with AutoDock Vina 1.2.5 using blind docking across the entire receptor. For OR10G4, the grid box was centered at (2.45, 3.93, 2.13) Å and measured 75.65 × 56.37 × 68.23 Å; for OR5M3, it was centered at (1.61, −4.26, 1.05) Å and measured 75.72 × 47.48 × 64.17 Å. Each calculation was independently repeated 10 times using different random seeds. The top-ranked poses were reproducible across runs, with a heavy-atom RMSD of 0 Å. Binary systems were docked using AutoDock Vina’s multi-ligand functionality, with both ligand PDBQT files supplied simultaneously and sampled within the same search space. The reported binary score represents the total score for the two-ligand complex, including receptor–ligand interactions for both ligands and interligand interactions. Binding poses were visualized in PyMOL (Schrödinger, LLC.,New York, NY, USA), and key noncovalent interactions, including hydrogen bonds and hydrophobic contacts, were systematically analyzed using LigPlot+ v2.3 (EMBL-EBI, Cambridge, UK).

### 2.7. Data Analysis

Correspondence analysis (CA) was applied to the complete product-by-attribute citation-frequency matrix. Sensory descriptors were retained before the volatile–sensory association analysis according to their contribution to product differentiation, redundancy with other descriptors, and chemical interpretability. Product-level Pearson correlation coefficients were then calculated between each volatile compound and each retained sensory descriptor. The two-sided *p* values for the complete family of correlations were adjusted jointly using the Benjamini–Hochberg procedure, with (*p_BH_* < 0.05) considered statistically significant. Because the sensory data consisted of bounded citation frequencies with tied values, Spearman rank correlations were also calculated as a sensitivity analysis and adjusted separately using the same procedure. Associations were considered robust when both methods produced coefficients in the same direction and (*p_BH_* < 0.05). For heatmap visualization, Pearson-correlation profiles were hierarchically clustered using Euclidean distance and average linkage.

## 3. Results and Discussion

### 3.1. Sensory Evaluation of Soy Sauce Samples

**Figure 1:**
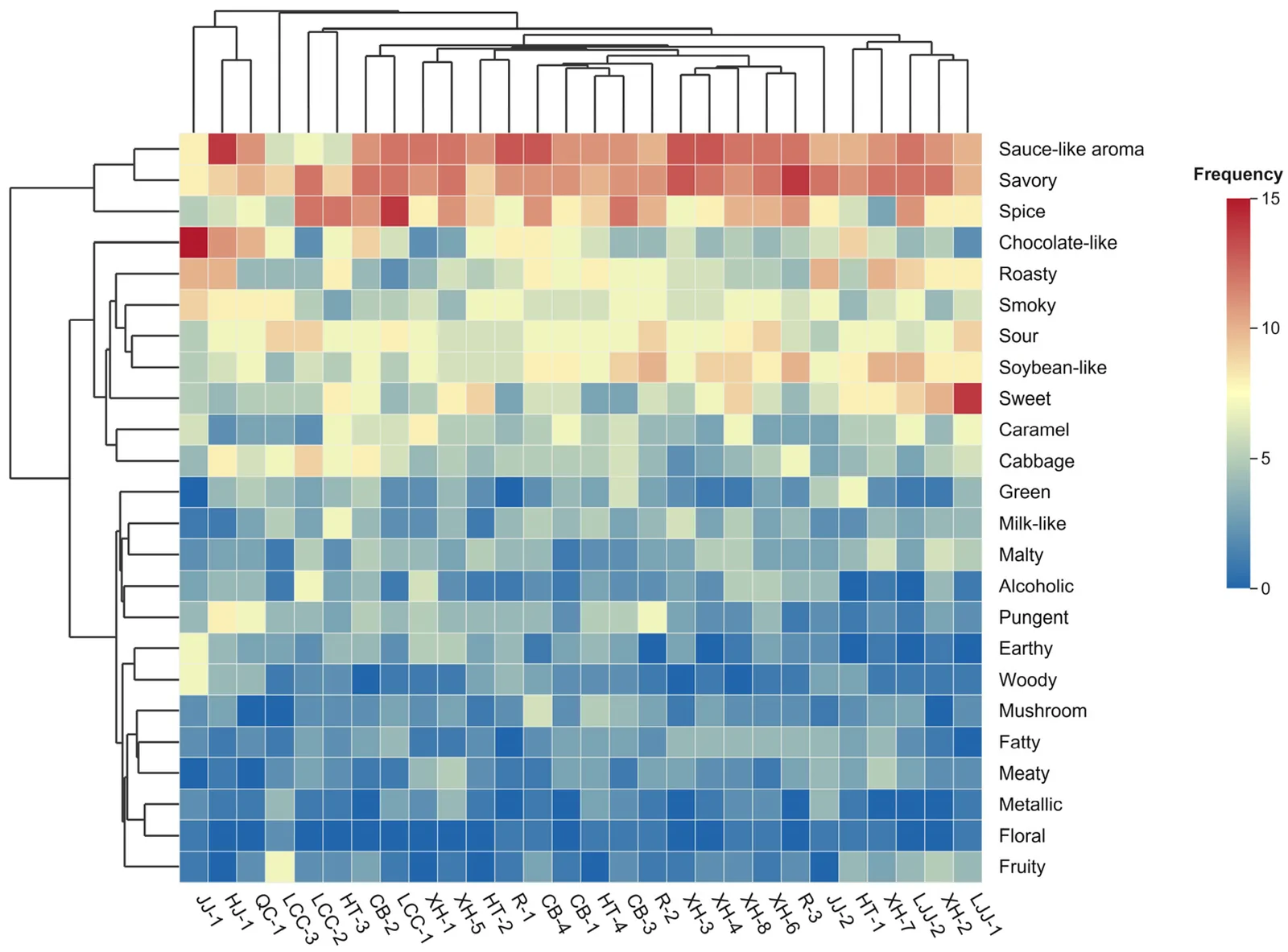
Hierarchical clustering heatmap of the complete CATA citation-frequency matrix.

Citation-frequency patterns for 24 CATA sensory descriptors across 28 soy sauce samples were examined descriptively. Because only aggregated citation frequencies were available, assessor-level binary responses could not be reconstructed. Cochran’s Q test was therefore not performed, and the observed differences should not be interpreted as inferentially significant. As shown in Figure 1, savory, sauce-like, soybean-like, and spicy were frequently cited across the samples. Savory (11.04 ± 1.37) and sauce-like aroma (10.89 ± 2.02) showed the highest mean citation frequencies and relatively low coefficients of variation (12.4% and 18.6%, respectively), suggesting that they represented broadly shared characteristics of the soy sauce samples. This pattern may be associated with protein degradation and the formation of Maillard reaction products during high-salt, dilute-state fermentation [23]. Spicy (8.82 ± 2.57) and soybean-like (7.36 ± 1.68) formed a secondary group, whereas sour (7.11 ± 1.13) and roasty (6.43 ± 2.23) were cited at moderate frequencies.

In contrast, floral (0.50 ± 0.58), metallic (1.36 ± 1.28), woody (1.89 ± 1.55), and fruity (1.96 ± 1.69) were infrequently cited overall. The high coefficients of variation observed for floral (115.5%) and fruity (86.0%) should be interpreted cautiously because their mean frequencies were very low. Nevertheless, a low overall frequency does not necessarily indicate an absence of discriminatory value, because an attribute cited primarily for a small subset of products may still contribute to product differentiation. Several samples displayed distinctive descriptive patterns. HT-3 had citation frequencies of 7 for both milk-like and cabbage-like, while LCC-1 showed the highest frequency for spicy (14), together with high frequencies for savory and sauce-like aroma (12 each). R-3 showed the highest frequency for savory (14), accompanied by soybean-like (10) and spicy (11). XH-2 was distinguished by a comparatively high frequency for sweet (10). These examples describe product-level citation patterns but do not establish statistically significant differences among samples or within-assessor co-occurrence of attributes.

Before the volatile–sensory association analysis, CA-guided reduction of the complete 28-product × 24-attribute matrix retained 13 descriptors: alcoholic, meaty, savory, malty, chocolate-like, woody, roasty, caramel, mushroom, spicy, green, smoky, and metallic. These descriptors were retained because they contributed to product differentiation while limiting redundancy among sensory terms. Although sauce-like aroma was frequently cited, it was excluded because it represented a broadly shared, holistic background characteristic with limited discriminatory contribution in the CA configuration. Conversely, some less frequently cited descriptors, including woody and metallic, were retained because their distributions contributed to differentiation among particular products. Overall, the descriptive CATA results indicated a shared sensory background dominated by savory, sauce-like, soybean-like, and spicy attributes, together with secondary attributes that differentiated particular products. Because assessor-level binary data were unavailable, these patterns are reported descriptively and are not used to infer statistically significant product differences, sensory intensity, common biochemical pathways, or causal effects of processing conditions.

### 3.2. Overview of Volatile Compounds in Soy Sauce

**Figure 2:**
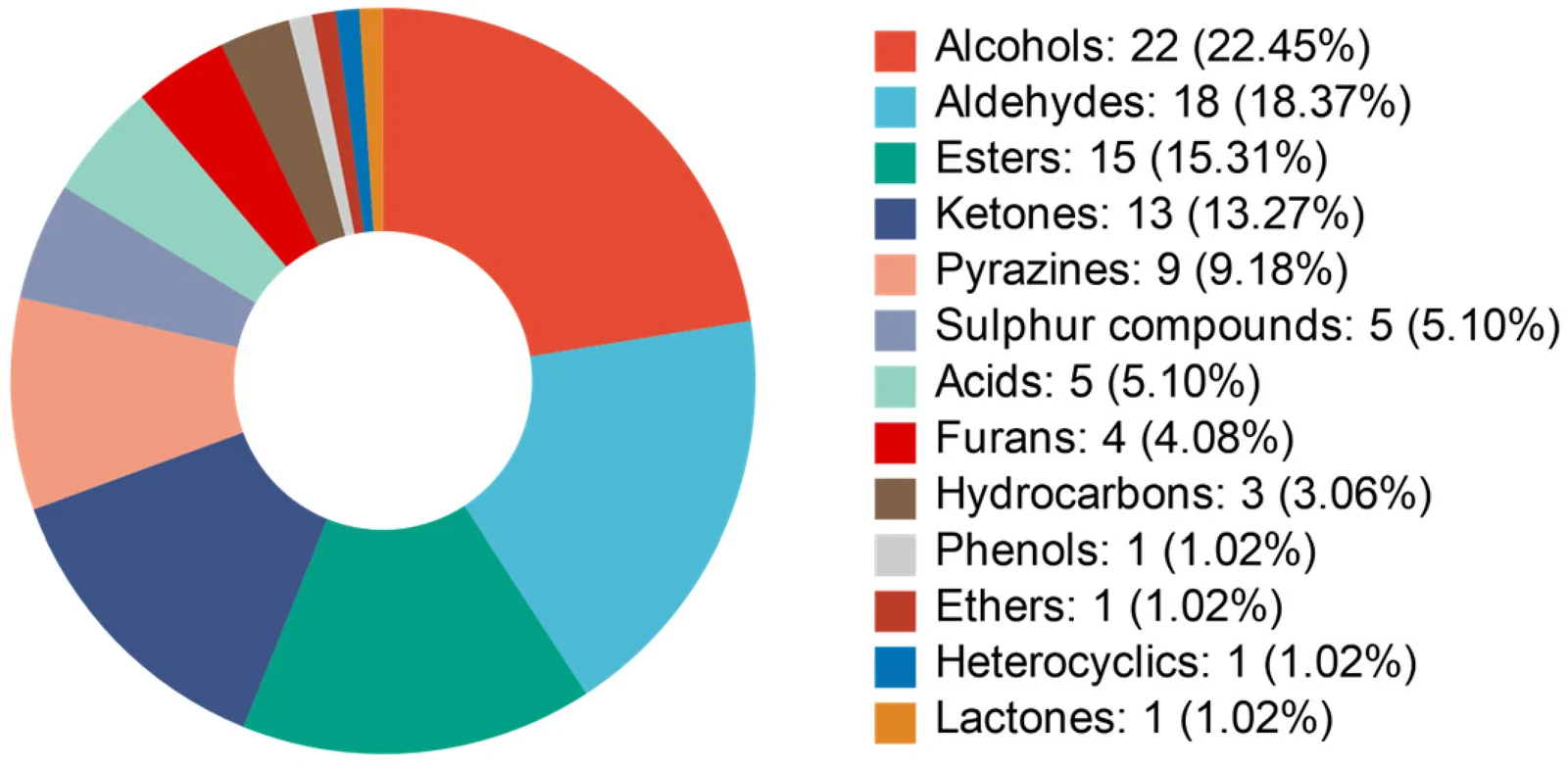
Distribution of the 99 detected volatile compounds among 14 chemical classes.

A total of 99 volatile compounds belonging to 14 chemical classes—including alcohols, aldehydes, esters, ketones, pyrazines, sulfur compounds, acids, furans, hydrocarbons, phenols, ethers, heterocyclic compounds, lactones, and amines—were detected across the 28 soy sauce samples by HS-GC-IMS. Alcohols were the largest class (*n* = 22, 22.22%), followed by aldehydes (*n* = 18, 18.18%), esters (*n* = 15, 15.15%), ketones (*n* = 13, 13.13%), and pyrazines (*n* = 9, 9.09%) (Figure 2). Sulfur compounds and acids each included five compounds (5.05%); the remaining classes occurred in smaller numbers. Aniline was counted as the single amine, which reconciles the class totals with the 99 analyte columns used in the correlation analysis. This class distribution is consistent with the established roles of yeast metabolism, amino-acid degradation, and Maillard chemistry in soy-sauce volatile formation [2,24].

**Figure 3:**
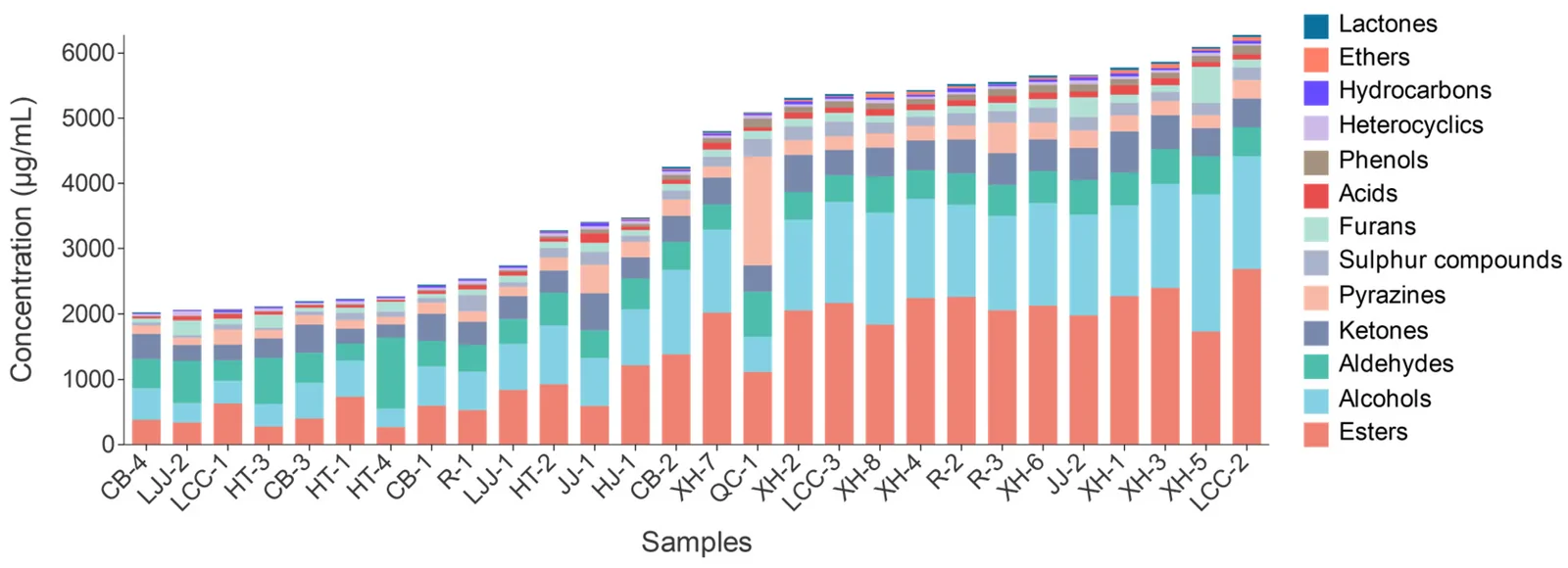
Stacked bar chart of compound concentrations in soy sauce samples.

Although alcohols were the most abundant class in number, esters accounted for the largest proportion of the total volatile concentration (38,111.77 μg/mL, 33.15% of all detected volatiles) (Figure 3). Alcohols ranked second (29,293.16 μg/mL, 25.48%), followed by aldehydes (13,856.11 μg/mL, 12.05%), ketones (11,459.36 μg/mL, 9.97%), pyrazines (7451.38 μg/mL, 6.48%), sulfur compounds (4066.44 μg/mL, 3.54%), furans (3805.67 μg/mL, 3.31%), acids (2128.75 μg/mL, 1.85%), and phenols (1690.60 μg/mL, 1.47%). Heterocyclic compounds, ethers, hydrocarbons, and lactones each contributed less than 1% of the total volatile content.

Ethyl acetate was the most abundant ester (25,190.38 µg/mL), followed by ethyl 2-hydroxypropanoate and ethyl propanoate. Among alcohols, isopentyl alcohol had the highest measured abundance (9352.37 µg/mL), followed by (Z)-3-hexenol, benzyl alcohol, and 1-octen-3-ol. Other abundant detected volatiles included 2-methylbutanal among aldehydes, 2-butanone among ketones, trimethylpyrazine among pyrazines, 2-methyl-3-furanthiol among sulfur compounds, 5-methyl-2(3H)-furanone among furans, 3-methylvaleric acid among acids, and guaiacol among phenols. Because these values were semiquantitative abundance estimates rather than aroma-impact measurements, abundance alone was not taken as evidence of sensory contribution.

Considerable differences in the total volatile concentrations were observed among the 28 soy sauce samples, ranging from 2024.56 to 6279.59 μg/mL, representing approximately a threefold variation. LCC-2 had the highest total volatile concentration (6279.59 μg/mL), followed by XH-5, XH-3, and XH-1, whereas CB-4, LJJ-2, and LCC-1 had the lowest levels. Similar variation was observed across individual chemical classes. Ester concentrations ranged from 268.59 μg/mL in HT-4 to 2690.03 μg/mL in LCC-2, while alcohols varied from 286.26 μg/mL (HT-4) to 2090.69 μg/mL (XH-5). In contrast, HT-4 contained the highest aldehyde concentration (1089.73 μg/mL), primarily due to its exceptionally high level of 2-methylbutanal, suggesting more extensive amino acid degradation and/or thermal reactions. Pyrazines were particularly enriched in QC-1, where their total concentration (1664.50 μg/mL) was substantially higher than the sample average, largely due to the accumulation of trimethylpyrazine. This enrichment may reflect differences in Maillard reaction intensity or fermentation conditions.

Overall, the volatile profiles of the samples were dominated by esters, alcohols, and aldehydes, which together accounted for 70.68% of the total volatile concentration. Ethyl acetate, isopentyl alcohol, and 2-methylbutanal were the most abundant compounds within their respective chemical classes. Although pyrazines and sulfur compounds were present at relatively lower concentrations, their low odor thresholds suggest that they contribute disproportionately to the characteristic roasted, nutty, and meaty aroma notes of soy sauce [25]. The pronounced differences in the abundance and composition of these major compound classes among the samples likely reflect variations in raw materials, fermentation conditions, and post-fermentation processing, providing a chemical basis for differentiating soy sauce products and supporting subsequent quality evaluation and process optimization.

### 3.3. Analysis of the Correlation Between Volatile Compounds and Sensory Attributes

**Figure 4:**
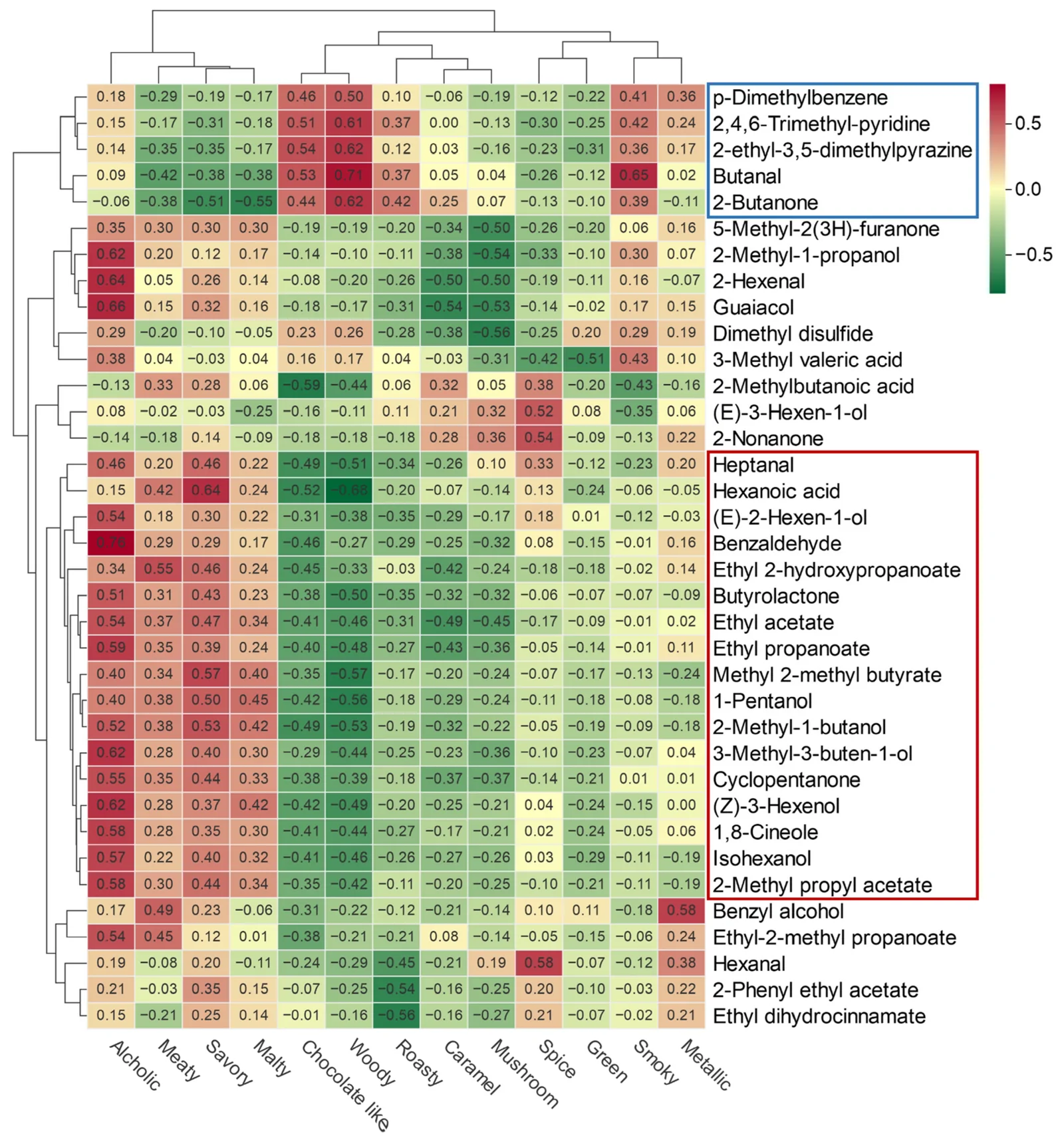
Pearson correlation analysis between volatile compounds and sensory attributes (|r| > 0.5, *p* < 0.05). Red squares indicate positive correlations, and green squares indicate negative correlations.

To further investigate the relationships between volatile compounds and the sensory characteristics of soy sauce, Pearson correlation analyses were performed between the concentrations of 99 volatile compounds in the samples and the scores of 13 prominent sensory attributes. As shown in Figure 4, the correlation network revealed two contrasting patterns of association. In general, compounds positively correlated with the alcoholic, savory, and malty attributes tended to be negatively correlated with the woody and chocolate-like attributes, suggesting that these sensory dimensions were associated with distinct groups of volatile compounds.

Among the compounds showing strong correlations (|r| > 0.5), one group (highlighted in red) was positively correlated with the alcoholic, savory, and malty attributes but negatively correlated with the woody and chocolate-like attributes. For example, hexanoic acid was positively correlated with the savory attribute (r = 0.64) but negatively correlated with the woody (r = −0.68) and chocolate-like (r = −0.52) attributes. Similarly, methyl 2-methylbutyrate was positively correlated with the savory attribute (r = 0.57) but negatively correlated with the woody attribute (r = −0.57). Similar correlation patterns were observed for 1-pentanol and 2-methyl-1-butanol. Most of these compounds belong to the chemical classes of fatty acids, branched-chain alcohols, and short-chain esters [19,26], which are commonly formed through amino acid and lipid metabolism during fermentation [24]. Their positive associations with the savory and malty attributes suggest that these compounds may contribute to the characteristic fermented aroma of soy sauce.

In contrast, another group of compounds (highlighted in blue) was positively correlated with the woody and chocolate-like attributes but negatively correlated with the savory and malty attributes. Representative compounds included 2-butanone, butanal, 2,4,6-trimethylpyridine, and 2-ethyl-3,5-dimethylpyrazine. These compounds mainly comprise ketones, aldehydes, and alkylpyrazines and may be formed through pathways such as the Maillard reaction, Strecker degradation, and lipid oxidation [27]. Their positive correlations with the woody and chocolate-like attributes suggest that they may be associated with the roasted aroma characteristics of soy sauce.

Taken together, these results indicate that the sensory attributes of soy sauce are associated with two major groups of volatile compounds. The first group is primarily associated with fermented characteristics represented by savory, alcoholic, and malty notes, whereas the second group is more closely associated with roasted characteristics, including woody and chocolate-like notes. These contrasting correlation patterns suggest that differences in the relative abundance of various classes of volatile compounds may contribute to the balance between the fermented and roasted aroma characteristics of soy sauce. However, correlation analysis alone cannot establish causal relationships. Therefore, the contributions of individual compounds to aroma perception should be further verified through aroma recombination and omission experiments or GC–O analysis.

### 3.4. Experiment on the Addition of Aroma Compounds

Candidate aroma compounds were added individually to the selected soy sauce matrices to evaluate their effects on five target attributes: savory, smoky, malty, meaty, and alcoholic notes.

Using the analytical approach described above, 15 candidate compounds were selected: butanal, 2,4,6-trimethylpyridine, 2-ethyl-3,5-dimethylpyrazine, heptanal, (E)-2-hexen-1-ol, benzaldehyde, (Z)-3-hexenol, 1-pentanol, 2-methyl-1-butanol, 3-methyl-3-buten-1-ol, isohexanol, ethyl propanoate, 2-methylpropyl acetate, methyl 2-methylbutyrate, and ethyl acetate. To experimentally evaluate the associations suggested by the Pearson correlation analysis, selected volatile compounds showing strong positive correlations with specific sensory attributes were individually added to representative soy sauce samples. The resulting changes in sensory characteristics were subsequently assessed by sensory evaluation (Table 2).

A total of 15 candidate compounds: butanal, 2,4,6-trimethylpyridine, 2-ethyl-3,5-dimethylpyrazine, heptanal, (E)-2-hexen-1-ol, benzaldehyde, (Z)-3-hexenol, 1-pentanol, 2-methyl-1-butanol, 3-methyl-3-buten-1-ol, isohexanol, ethyl propanoate, 2-methylpropyl acetate, methyl 2-methylbutyrate, and ethyl acetate were selected using the analytical methodology outlined above. To further validate the relationships identified by the Pearson correlation analysis, selected volatile compounds that exhibited significant positive correlations with specific sensory attributes were individually added to representative soy sauce samples and subsequently evaluated by sensory evaluation (Table 3).

The addition experiments generally supported the correlation analysis (Table 3), although not all compounds produced the expected sensory responses. Under the present experimental conditions, the addition of benzaldehyde and ethyl propanoate significantly enhanced the savory attribute, suggesting that these compounds contribute positively to the perception of savory aroma in soy sauce. Meanwhile, supplementation with 2-ethyl-3,5-dimethylpyrazine significantly increased the alcoholic attribute, indicating that this compound is closely associated with alcoholic aroma perception. Similarly, methyl 2-methylbutyrate significantly enhanced smoky, meaty, and alcoholic attributes, whereas ethyl acetate significantly increased malty, meaty, and alcoholic aromas. These results indicate that individual volatile compounds may simultaneously influence multiple sensory attributes [28], reflecting the multidimensional contribution of aroma compounds to soy sauce flavor. The results described above reflect sensory responses influenced by both the matrix and the concentration of the added compounds.

Not all compounds exhibiting positive correlations in the correlation analysis produced significant aroma enhancement when evaluated individually. Under the present experimental conditions, Several compounds, including butanal, heptanal, (Z)-3-hexenol, and 2-methyl propyl acetate, significantly reduced the intensity of one or more sensory attributes after addition. For example, butanal significantly decreased alcoholic aroma, whereas heptanal reduced savory, malty, and meaty attributes. These findings indicate that the sensory effects of individual compounds do not always correspond directly to their statistical correlations and suggest that aroma perception in soy sauce depends on interactions within the complex volatile matrix.

Overall, the addition experiments partially validated the Pearson correlation results. Various compounds—including benzaldehyde, ethyl propionate, 2-ethyl-3,5-dimethylpyrazine, methyl 2-methylbutyrate, and ethyl acetate—enhanced at least one target attribute, although several also suppressed other attributes, indicating attribute-specific and matrix-dependent effects. Nevertheless, because aroma perception is governed by complex interactions among multiple volatile compounds, the sensory contribution of these compounds should be further verified using aroma recombination, omission experiments, or GC–O analysis.

### 3.5. Discussion of Molecular Docking Results

#### 3.5.1. Predicted Binding Affinities of Odorants with OR10G4 and OR5M3

Molecular docking was performed to generate structural models of individual odorants and binary odorant combinations within the binding cavities of OR10G4 and OR5M3, and the resulting docking scores are summarized in Table 4. Although a lower Vina score generally indicates a more energetically favourable predicted binding pose for comparable systems, the score of a binary system represents the total score of the two-ligand complex and is therefore not directly comparable with that of a single ligand. Because the scoring function aggregates ligand–receptor interaction terms, a more negative binary score cannot by itself be interpreted as evidence of cooperative, synergistic, or stronger binding, nor does molecular docking directly demonstrate receptor activation or sensory perception [29]. Guaiacol alone exhibited a predicted docking score of −5.66 kcal·mol^−1^ with OR10G4, whereas the total scores of the guaiacol-containing binary systems ranged from −8.27 to −10.10 kcal·mol^−1^. Among these binary systems, guaiacol–2-ethyl-3,5-dimethylpyrazine showed the lowest total score (−10.10 kcal·mol^−1^), followed by guaiacol–methyl 2-methylbutyrate (−9.98 kcal·mol^−1^), while guaiacol–butanal exhibited a comparatively less negative score (−8.27 kcal·mol^−1^). These binary scores are presented only for qualitative comparison among systems of broadly similar size and suggest that the presence of a second odorant may alter the predicted binding conformation and interaction network of guaiacol within the OR10G4 cavity. Accordingly, the binary docking results should be regarded as exploratory co-binding conformational models rather than evidence of enhanced or synergistic receptor binding.

A similar trend was observed for homofuraneol docking with OR5M3. The predicted binding energy of homofuraneol alone was −6.50 kcal·mol^−1^, whereas all binary ligand systems exhibited substantially lower docking scores (−10.16 to −13.32 kcal·mol^−1^). The homofuraneol–benzaldehyde system had the lowest predicted binding energy (−13.32 kcal·mol^−1^), followed by the homofuraneol–heptanal (−11.45 kcal·mol^−1^) and homofuraneol–2-methyl propyl acetate (−11.28 kcal·mol^−1^) systems. Binary complexes containing ethyl propanoate (−10.16 kcal·mol^−1^) and (Z)-3-hexenol (−10.41 kcal·mol^−1^) also exhibited more favourable docking scores than homofuraneol alone.

Overall, the incorporation of a second odorant consistently resulted in lower predicted docking energies than the corresponding single-ligand systems. These findings suggest that binary odorant systems may occupy the receptor binding pocket in energetically more favorable conformations than individual odorants. However, it should be emphasized that docking scores represent computational predictions of binding stability rather than direct measurements of receptor activation or aroma perception. Therefore, these results should be interpreted as providing a possible structural explanation for the sensory enhancement observed in the aroma addition experiments rather than direct evidence of receptor activation.

#### 3.5.2. Predicted Binding Modes of Guaiacol and Homofuraneol with OR10G4 and OR5M3

**Figure 5:**
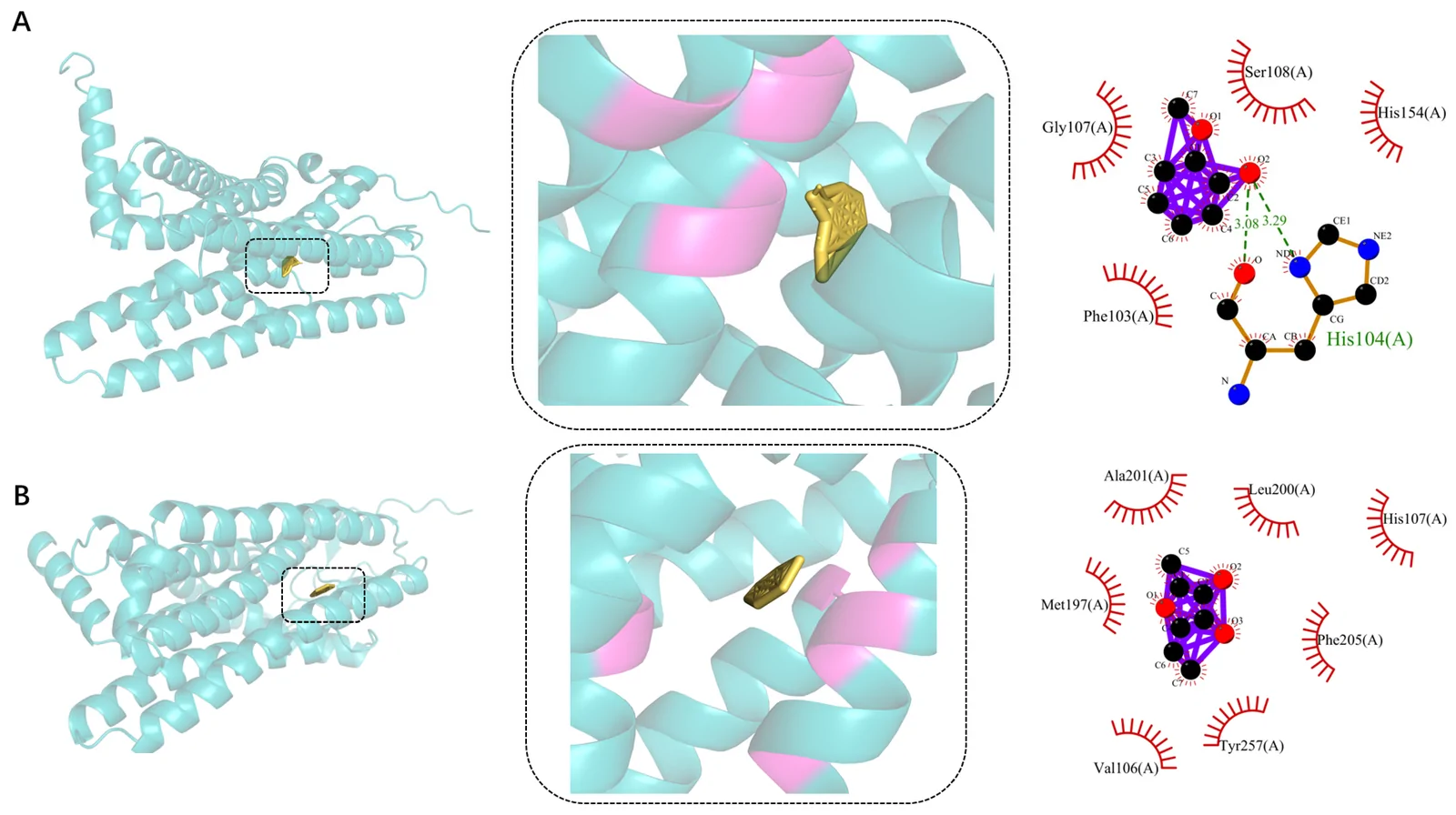
Binding modes of guaiacol and homofuraneol with OR10G4 and OR5M3. (**A**) Guaiacol with OR10G4; (**B**) homofuraneol with OR5M3.

To further investigate the structural basis underlying the differences in docking scores, the optimal docking conformations were analysed using LigPlot+ [30], which identifies hydrogen bonds and hydrophobic contacts between ligands and receptor residues (Figure 5) [31]. Hydrogen bonds contribute to ligand orientation and binding specificity, whereas hydrophobic interactions are generally regarded as the major driving force stabilizing odorant binding within the hydrophobic cavity of olfactory receptors [32].

#### 3.5.3. Predicted Interaction Pattern of Guaiacol with OR10G4

The predicted interaction residues between guaiacol (or binary ligand systems) and OR10G4 are summarized in Table 5. Guaiacol alone was predicted to interact with OR10G4 through a single hydrogen bond involving His104, together with hydrophobic contacts formed by Phe103, Gly107, Ser108, and His154. This relatively simple interaction network corresponded to the moderate docking score observed for the single-ligand model.

Upon introduction of a second odorant, both the hydrogen-bonding pattern and hydrophobic interaction network were altered substantially. In the guaiacol–methyl 2-methylbutyrate system, guaiacol was predicted to form a hydrogen bond with Pro182, whereas methyl 2-methylbutyrate formed two additional hydrogen bonds with His154 and Asp201. In addition, guaiacol formed additional hydrophobic contacts with Gln172, Ile173, Gln174, Lys185, and Leu186, while methyl 2-methylbutyrate interacted extensively with residues near the orthosteric binding pocket, including Phe103, His104, Gly107, Ser108, Cys111, and Val205. Compared with the single-ligand model, the binary co-binding model exhibited a larger predicted receptor–ligand contact surface [33]. We note, however, that this observation describes the predicted pose geometry only and does not constitute evidence of stronger binding.

The guaiacol–2-ethyl-3,5-dimethylpyrazine system had the lowest docking score among all OR10G4 models. Although 2-ethyl-3,5-dimethylpyrazine did not form predicted hydrogen bonds, it established extensive hydrophobic contacts with Pro181, Val195, Ile198, Phe199, Ile202, and Tyr258. Together with the additional hydrophobic contacts formed by guaiacol, these interactions generated a dense hydrophobic network surrounding the receptor binding pocket. Since odorant binding in Class A GPCRs is generally dominated by hydrophobic interactions [34], this extensive hydrophobic complementarity may account for the more favourable predicted binding conformation of this binary ligand system.

In contrast, butanal generated a relatively smaller interaction network. Although guaiacol formed three hydrogen bonds with His104, His154, and Asp201, butanal itself formed only hydrophobic contacts with Phe112, Ile204, Val205, Gly208, and Cys209. The more limited hydrophobic interface predicted for the guaiacol–butanal model suggests a sparser interaction network in this system relative to the other binary models; given the caveats above, no conclusion about relative binding affinity is drawn. Overall, the exploratory models suggest that the presence of a second odorant reorganizes the predicted interaction network—particularly the hydrophobic contacts—within the receptor cavity, an observation consistent with the generally hydrophobic character of olfactory receptor binding pockets.

#### 3.5.4. Binding Patterns of Homofuraneol and Its Binary-Ligand System with OR5M3

The predicted interaction residues between homofuraneol (or binary ligand systems) and OR5M3 are summarized in Table 6. Unlike guaiacol, homofuraneol alone did not form hydrogen bonds with OR5M3. Instead, binding was predicted to rely entirely on hydrophobic interactions involving Val106, His107, Met197, Leu200, Ala201, Phe205, and Tyr257. This result suggests that hydrophobic interactions play a dominant role in stabilizing the homofuraneol–OR5M3 complex.

Among the binary ligand systems, hydrogen bonding was detected only in the homofuraneol–heptanal model, where homofuraneol formed a single hydrogen bond with Arg260. In all remaining systems, no hydrogen bonds were predicted, indicating that the observed lower docking scores were accompanied by changes in the predicted hydrophobic-contact network. The binary ligand systems exhibited distinct hydrophobic interaction patterns. In the homofuraneol–benzaldehyde model, homofuraneol interacted with Ile103, His107, Asp179, Met197, Leu200, Ala201, and Tyr257, whereas benzaldehyde formed additional contacts with Phe102, Ile103, Val106, His107, Phe205, and Tyr257. Notably, several residues—including His107 and Tyr257—were shared between the two ligands, resulting in a more continuous hydrophobic interaction network within the receptor binding cavity. This extensive contact network was associated with the lowest docking score among all OR5M3 systems.

The heptanal-containing system also had a relatively favourable docking score. In addition to the hydrogen bond formed by homofuraneol with Arg260, heptanal formed hydrophobic interactions with Phe102, Val106, Phe205, and Tyr257. The coexistence of hydrogen-bonding and hydrophobic interactions may account for the relatively stable predicted binding conformation observed in this model. For the remaining binary systems containing ethyl propanoate, (Z)-3-hexenol, and 2-methyl propyl acetate, the second ligand interacted with distinct regions of the OR5M3 binding pocket, resulting in different hydrophobic contact patterns. Accordingly, the predicted binding orientation of homofuraneol also changed, leading to increased receptor–ligand contact areas and more favourable docking scores than those of the single-ligand model.

Taken together, the docking analysis suggests that co-present odorants may reorganize the predicted interaction network within the receptor binding cavity, particularly through altered hydrophobic contact patterns. These computational models are hypothesis-generating: they do not measure binding affinity, receptor activation, or perception, and the apparent numerical differences between single- and dual-ligand scores reflect, at least in part, the larger atom count of the binary systems. Whether the predicted co-binding modes translate into receptor activation and perceptual modulation requires validation using functional olfactory receptor assays, such as calcium imaging or luciferase reporter experiments [35].

The molecular docking results consistently showed that binary odorant systems exhibited more favorable predicted binding conformations than the corresponding single-odorant systems. Rather than simply increasing the number of hydrogen bonds, the coexisting odorants primarily reshaped the interaction network within the receptor binding pocket by expanding hydrophobic contacts and, in some cases, introducing additional hydrogen-bonding interactions. Such structural rearrangements were accompanied by lower predicted docking energies and may facilitate more stable receptor–ligand complexes. Importantly, these computational findings are consistent with the sensory enhancement observed in the aroma addition experiments, suggesting that odor–odor interactions may influence aroma perception, at least in part, through changes in receptor binding. However, these conclusions should be interpreted with caution because molecular docking predicts only potential binding modes and cannot directly demonstrate receptor activation or sensory responses. Future studies combining receptor-based functional assays and aroma recombination experiments will be necessary to further verify the proposed molecular mechanism.

## 4. Conclusions

This study integrated HS–GC–IMS volatile profiling, CATA sensory evaluation, product-level correlation analysis, aroma addition experiments, and molecular docking to examine aroma differences among 28 commercial soy sauces. A total of 99 volatile compounds from 14 chemical classes were detected, with total semiquantitative concentrations ranging from 2024.56 to 6279.59 μg/mL. Esters, alcohols, and aldehydes together accounted for 70.68% of the total volatile content. CATA citation frequencies indicated a common sensory background dominated by savory, sauce-like, soybean-like, and spicy attributes, while 13 of the 24 descriptors were retained for product differentiation. Exploratory Pearson analysis revealed two contrasting association patterns: fatty acids, branched-chain alcohols, and selected esters were mainly associated with alcoholic, savory, and malty attributes, whereas several aldehydes, ketones, and alkylpyrazines were associated with woody, chocolate-like, and roasted characteristics.

Single-compound addition experiments involving 15 candidates only partially reproduced these product-level associations. Benzaldehyde and ethyl propanoate enhanced savory perception; 2-ethyl-3,5-dimethylpyrazine enhanced the alcoholic attribute; methyl 2-methylbutyrate enhanced smoky, meaty, and alcoholic attributes; and ethyl acetate enhanced malty, meaty, and alcoholic attributes. Other compounds produced opposing or nonsignificant responses, demonstrating that correlation alone cannot establish sensory causality and that sensory effects depend on compound concentration and matrix composition. Dual-ligand docking predicted altered ligand orientations and expanded hydrophobic-contact networks within OR10G4 and OR5M3. Among the binary models, guaiacol–2-ethyl-3,5-dimethylpyrazine and homofuraneol–benzaldehyde yielded the lowest total docking scores for OR10G4 and OR5M3, respectively.

The novelty of this study lies in the construction of a multi-level framework to dissect aroma differences in commercial soy sauce. HS-GC-IMS and CATA were combined to establish product-level associations between volatile compounds and sensory attributes. Aroma addition experiments in real soy sauce matrix were then carried out to distinguish simple statistical correlation from actual sensory contribution. Dual-ligand molecular docking was further used to predict the co-binding characteristics of odorants on olfactory receptors OR10G4 and OR5M3 for mechanistic exploration. However, multi-ligand scores cannot be directly compared with single-ligand scores or interpreted as evidence of synergistic binding or receptor activation. Overall, this workflow provides a practical approach for screening candidate aroma compounds, verifying their matrix-dependent sensory effects, and generating testable hypotheses concerning odorant–receptor interactions.

## Figures and Tables

**Table 1:** Information on the 28 commercial soy sauce samples.

Soy Sauce Names	Codes
Xinhe Low-Salt Original Brew Soy Sauce	XH-1
Xinhe Low-Salt Oyster Flavored Soy Sauce	XH-2
Japanese Light Soy Sauce	R-1
Jiajia Soy Sauce	JJ-1
Lao Caichen Soy Sauce for Cold Dishes	LCC-1
Xinhe Naturally Fermented Soy Sauce	XH-3
Chubang Premium Light Soy Sauce	CB-1
Haitian Reduced-Salt Soy Sauce	HT-1
Chubang Low-Salt Soy Sauce	CB-2
Chubang Delicious Fresh Soy Sauce	CB-3
Chubang Gold Premium Light Soy Sauce	CB-4
Xinhe Organic Soy Sauce	XH-4
Xinhe Liuyue Tian Braising Dark Soy Sauce	XH-5
Haitian Seafood Flavored Soy Sauce	HT-2
Xinhe Organic Soy Sauce for Children	XH-8
Jiajia Premium Umami Soy Sauce	JJ-2
Xinhe Kombu Umami Soy Sauce	XH-6
Xinhe Premium Liuyuexian Soy Sauce	XH-7
Haitian Straw Mushroom Dark Soy Sauce	HT-3
Lee Kum Kee Less Salt Light Soy Sauce	LJJ-1
Lao Caichen Mild Premium Soy Sauce	LCC-2
Lee Kum Kee Selected Dark Soy Sauce	LJJ-2
Haitian Straw Mushroom Braising Dark Soy Sauce	HT-4
Hamada Japanese Soy Sauce	R-2
Haoji Wooden Barrel Fermented Soy Sauce	HJ-1
Lao Caichen No.1 Premium Fresh Soy Sauce	LCC-3
Maggi Liquid Seasoning	QC-1
Japanese Pure Brewed Black Bean Soy Sauce	R-3

**Table 2:** Addition amount of the candidate aroma compounds in the five soy sauce samples.

Compounds	Addition Amount ^a^				
	JJ-1 Original Sample	HT-3 Original Sample	CB-1 Original Sample	QC-1 Original Sample	HT-1 Original Sample
	Savory	Smoky	Malty	Meaty	Alcoholic
Butanal	-	781	-	-	209
2,4,6-Trimethyl-pyridine	-	638	55	11	55
2-Ethyl-3,5-dimethylpyrazine	-	320	10	-	30
Heptanal	20	-	20	-	30
(E)-2-Hexen-1-ol	51	31	41	296	174
Benzaldehyde M	92	-	61	-	376
(Z)-3-Hexenol	308	-	1375	1324	2452
1-Pentanol	406	81	487	568	487
2-Methyl-1-butanol	180	10	200	230	280
3-Methyl-3-buten-1-ol	-	48	60	36	120
Isohexanol	90	-	70	80	250
Ethyl propanoate	1782	187	1155	1188	2035
2-Methyl propyl acetate	180	-	216	204	552
Methyl 2-methyl butyrate	120	10	140	150	150
Ethyl acetate	10,914	2075	9110	8389	10,012

Note: “-“: No additives; ^a^: Each value denotes the mass of individual compound spiked into 10 mL of soy sauce (μg).

**Table 3:** Effects of candidate aroma compounds on the five target sensory attributes.

Added Compounds	Control Group 1	Control Group 2	Control Group 3	Control Group 4	Control Group 5
	JJ-1 Original Sample	HT-3 Original Sample	CB-1 Original Sample	QC-1 Original Sample	HT-1 Original Sample
	Savory	Smoky	Malty	Meaty	Alcoholic
Butanal		↓ **			↓ ***
2,4,6-Trimethyl-pyridine		-	-	-	-
2-Ethyl-3,5-dimethylpyrazine		↓ ***	↓ **		↑ **
Heptanal	↓ ***		↓ *	↓ **	-
(E)-2-Hexen-1-ol	-	-	-	-	-
Benzaldehyde	↑ **		↓ **		↓ **
(Z)-3-Hexenol	↓ ***		↓ ***	↓ ***	↓ ***
1-Pentanol	-	-	-	-	-
2-Methyl-1-butanol	-	-	-	-	-
3-Methyl-3-buten-1-ol		-	-	-	-
Isohexanol	-		-	-	-
Ethyl propanoate	↑ ***	-	↓ *	↓ **	↓ ***
2-Methyl propyl acetate	↓ ***		↓ ***	↓ **	↓ **
Methyl 2-methyl butyrate	-	↑ **	-	↑ *	↑ *
Ethyl acetate	↓ **	-	↑ *	↑ *	↑ **

Note: Blank, No additives; -, no significant difference; *, significant (*p* ≤ 0.05); **, highly significant (*p* ≤ 0.01); ***, very highly significant (*p* ≤ 0.001), ↑ and ↓ denote increases and decreases in sensory-attribute upon compound addition, respectively.

**Table 4:** Binding energies of odorants with olfactory receptors OR10G4 and OR5M3.

Odorizer	OR10G4 Binding Energy (kcal/mol)	OR5M3 Binding Energy (kcal/mol)
Guaiacol	−5.66	-
Guaiacol + Methyl 2-methylbutyrate	−9.98	-
Guaiacol + 2-ethyl-3,5-dimethylpyrazine	−10.10	-
Guaiacol + Butanal	−8.27	-
Homofuraneol	-	−6.50
Homofuraneol + Ethyl propanoate	-	−10.16
Homofuraneol + Benzaldehyde	-	−13.32
Homofuraneol + Heptanal	-	−11.45
Homofuraneol + (Z)-3-Hexenol	-	−10.41
Homofuraneol + 2-Methyl propyl acetate	-	−11.28

Note: “-“ indicates that docking for that receptor was not performed; OR, olfactory receptor.

**Table 5:** Binding sites of Odorants with OR10G4.

Odorizer	Hydrogen-Bonding Sites	Sites of Hydrophobic Interactions
Guaiacol	His104	Phe103, Gly107, Ser108, His154
Guaiacol + Methyl 2-methylbutyrate	Guaiacol: Pro182 Methyl 2-methylbutyrate: His154, Asp201	Guaiacol: Gln172, Ile173, Gln174, Lys185, Leu186 Methyl 2-methylbutyrate: Phe103, His104, Gly107, Ser108, Cys111, Val205
Guaiacol + 2-ethyl-3,5-dimethylpyrazine	Guaiacol: Pro182 2-ethyl-3,5-dimethylpyrazine: NA	Guaiacol: Gln172, Ile173, Gln174, Lys185, Leu186 2-ethyl-3,5-dimethylpyrazine: Pro181, Val195, Ile198, Phe199, Ile202, Tyr258
Guaiacol + Butanal	Guaiacol: His104, His154, Asp201 Butanal: NA	Guaiacol: Phe103, Gly107, Ser108 Butanal: Phe112, Ile204, Val205, Gly208, Cys209

Note: NA indicates that no hydrogen bonds were detected.

**Table 6:** Binding sites of Odorants with OR5M3.

Odorizer	Hydrogen-Bonding Sites	Sites of Hydrophobic Interactions
Homofuraneol	NA	Val106, His107, Met197, Leu200, Ala201, Phe205, Tyr257
Homofuraneol + Ethyl propanoate	Homofuraneol: NA Ethyl propanoate: NA	Homofuraneol: His107, Met 197, Leu200, Ala201, Tyr 257 Ethyl propanoate: Asn74, Lys78, Ile90, Gly94, Val97, Gln98
Homofuraneol + Benzaldehyde	Homofuraneol: NA Benzaldehyde: NA	Homofuraneol: Ile103, His107, Asp179, Met 197, Leu200, Ala201, Tyr 257 Benzaldehyde: Phe102, Ile103, Val106, His107, Phe 205, Tyr257
Homofuraneol + Heptanal	Homofuraneol: Arg260 Heptanal: NA	Homofuraneol: Glu194, Tyr195, Ile198 Heptanal: Phe102, Val106, Phe205, Tyr257
Homofuraneol + (Z)-3-Hexenol	Homofuraneol: NA (Z)-3-Hexenol: NA	Homofuraneol: Tyr216, Ile219, Leu220, Ile223, Phe236, Cys239, Leu243 (Z)-3-Hexenol: Phe102, Val106, Ala201, Phe205, Tyr257
Homofuraneol + 2-Methyl propyl acetate	Homofuraneol: NA 2-Methyl propyl acetate: NA	Homofuraneol: Phe111, Ala114, Ala115, Leu142, Pro146 2-Methyl propyl acetate: Phe102, Ile103, Val106, His107, Ala201, Phe205, Tyr257

Note: NA indicates that no hydrogen bonds were detected.

## Data Availability

The original contributions presented in this study are included in the article and Supplementary Materials. Further inquiries can be directed to the corresponding authors.

## Supplementary Materials

The following supporting information can be downloaded at: https://www.mdpi.com/article/10.3390/foods15193545/s1, Table S1: Key Aroma Compounds and Their Corresponding OAVs in Different Samples.
