# Supplementary material for: Volatile Chemistry and Aroma Differences in Commercial Soy Sauces: Sensory Links and Odorant–Receptor Interactions
Source: Foods. 2026 Oct 4;15(19):3545. doi: 10.3390/foods15193545 (PMC13665231; doi:10.3390/foods15193545)
Supplement: Supplementary file 1 [file foods-15-03545-s001.zip › foods-4494118-supplementary.pdf]

1.OAV values of some volatile compounds in the sample.

Table S1 Key Aroma Compounds and Their Corresponding OAVs in Different Samples

| Compound | Threshold (mg/kg) | OAV | | | | | | | | | |
|---|---|---|---|---|---|---|---|---|---|---|---|
| | | R-3 | XH-7 | XH-1 | JJ-1 | LCC-3 | HT-3 | CB-1 | QC-1 | LJJ-2 | HT-1 |
| Butanal | 0.009 | 6 | 57 | 70 | 124 | 66 | 46 | 80 | 76 | 67 | 49 |
| 2,4,6-Trimethyl-pyridine | 0.002 | 7 | 9 | 9 | 68 | 6 | 4 | 4 | 8 | 5 | 4 |
| 2-Ethyl-3,5-dimethylpyrazine | 0.001 | 5 | 4 | 6 | 35 | 5 | 3 | 3 | 11 | 3 | 3 |
| Heptanal | 0.003 | 5 | 5 | 5 | 3 | 5 | 3 | 3 | 2 | 2 | 2 |
| (E)-2-Hexen-1-ol | 0.1 | 95 | 33 | 19 | 5 | 13 | 2 | 2 | 4 | 2 | 2 |
| Benzaldehyde M | 0.35 | 25 | 20 | 47 | 16 | 29 | 16 | 14 | 22 | 16 | 9 |
| (Z)-3-Hexenol | 0.0039 | 68 | 151 | 267 | 37 | 110 | 60 | 13 | 19 | 67 | 22 |
| 1-Pentanol | 0.12 | 52 | 61 | 53 | 9 | 32 | 2 | 14 | 3 | 2 | 8 |
| 2-Methyl-1-butanol | 0.0159 | 21 | 27 | 30 | 3 | 11 | 2 | 7 | 3 | 1 | 2 |
| 3-Methyl-3-buten-1-ol | 0.547125 | 7 | 9 | 16 | 7 | 9 | 3 | 2 | 5 | 2 | 4 |
| Isohexanol | 0.82 | 10 | 9 | 27 | 1 | 8 | 1.06 | 2 | 1 | 1 | 1 |
| Ethyl propanoate | 0.01 | 200 | 129 | 217 | 22 | 267 | 3 | 13 | 10 | 3 | 14 |
| 2-Methyl propyl acetate | 0.025 | 19 | 22 | 56 | 1 | 4 | 2233 | 1 | 2 | 1 | 1 |
| Methyl 2-methyl butyrate | 0.00025 | 13 | 16 | 17 | 2 | 4 | 1 | 2 | 1 | <1 | 2 |
| Ethyl acetate | 0.005 | 1411 | 1746 | 1531 | 320 | 1402 | 112 | 433 | 910 | 184 | 531 |

Note, the odor threshold value was detected in water matrix.
